# Influence of Grain-Growth Inhibitors on Modified (Ba,Sr)(Sn,Ti)O_3_ for Electrocaloric Application

**DOI:** 10.3390/ma17051036

**Published:** 2024-02-23

**Authors:** Zhenglyu Li, Christian Molin, Sylvia E. Gebhardt

**Affiliations:** Fraunhofer Institute for Ceramic Technologies and Systems IKTS, Winterbergstrasse 28, 01277 Dresden, Germany; zhenglyu.li@ikts.fraunhofer.de (Z.L.); christian.molin@ikts.fraunhofer.de (C.M.)

**Keywords:** grain growth inhibitors, modification, electrocaloric

## Abstract

The paper reports on effect of grain-growth inhibitors MgO, Y_2_O_3_ and MnCO_3_ as well as Ca modification on the microstructure, dielectric, ferroelectric and electrocaloric (EC) properties of Ba_0.82_Sr_0.18_Sn_0.065_Ti_0.935_O_3_ (BSSnT). Furthermore, the effects of the sintering time and temperature on the microstructure and the electrical properties of the most promising material system Ba_0.62_Ca_0.20_Sr_0.18_Sn_0.065_Ti_0.935_O_3_ (BCSSnT-20) are investigated. Additions of MgO (*x*_MgO_ = 1%), Y_2_O_3_ (*x*_Y_2_O_3__ = 0.25%) and MnCO_3_ (*x*_MnCO_3__ = 1%) significantly decreased the mean grain size of BSSnT to 0.4 µm, 0.8 µm and 0.4 µm, respectively. Ba_0.62_Ca_0.20_Sr_0.18_Sn_0.065_Ti_0.935_O_3_ (BCSSnT-20) gained a homogeneous fine-grained microstructure with an average grain size of 1.5 µm, leading to a maximum electrocaloric temperature change |Δ*T*_EC_| of 0.49 K at 40 °C with a broad peak of |Δ*T*_EC_| > 0.33 K in the temperature range from 10 °C to 75 °C under an electric field change of 5 V µm^−1^. By increasing the sintering temperature of BCSSnT-20 from 1350 °C to 1425 °C, the grain size increased from 1.5 µm to 7.3 µm and the maximum electrocaloric temperature change |Δ*T*_EC_| increased from 0.15 K at 35 °C to 0.37 K at 20 °C under an electric field change of 2 V µm^−1^. Our results show that under all investigated material systems, BCSSnT-20 is the most promising candidate for future application in multilayer ceramic (MLC) components for EC cooling devices.

## 1. Introduction

The electrocaloric (EC) effect, defined as the reversible adiabatic temperature change or the isothermal entropy change of a dielectric material under the application of an electric field [1], is promising for the development of alternative cooling devices. To realize solid-state cooling based on the EC effect, various material systems have been explored so far. Besides lead-containing EC material systems such as PbZr_0.95_Ti_0.05_O_3_ (PZT) [2], PbSc_0.5_Ta_0.5_O_3_ (PST) [3] and (1 − x)Pb(Mg_1/3_Nb_2/3_)O_3_–xPbTiO_3_ (PMN-PT) [4], investigations on lead-free EC material systems based on ferroelectric BaTiO_3_ [5,6,7,8] as well as the relaxor ferroelectrics Bi_0.5_Na_0.5_TiO_3_ [9,10,11] and K_0.5_Na_0.5_NbO_3_ [12,13] have also been widely reported.

Bulk ceramics, thin films and multilayer ceramics (MLC) are typical component structures for EC cooling devices. Among them, MLC components are the most suitable because of their increased cooling capacity compared to thin films and their higher dielectric breakdown strength compared to bulk ceramics. In our previous work, we successfully implemented the lead-based 0.92Pb(Mg_1/3_Nb_2/3_)O_3_–0.08PbTiO_3_ (PMN-8PT) material into MLC components to allow for the application of higher electrical loads compared to bulk ceramics and thus achieve higher EC temperature changes [14]. However, the fabrication of MLC components based on our developed lead-free material system Ba_0.82_Sr_0.18_Sn_0.065_Ti_0.935_O_3_ (BSSnT) [15] turned out to be challenging due to the discontinuous grain growth resulting in a mean grain size of around 40 µm, which causes electrical conductivity paths between the inner electrodes. To inhibit the grain growth, we added MgO to BSSnT in our previous work and achieved a substantial decrease in grain size from 40 µm to 0.4 µm [16]. Simultaneously, this reduction in grain size caused a substantial decrease in the EC temperature change. In our current study, we investigate alternative grain-growth inhibitors, which exhibit a gentler effect on grain-growth suppression, so as to avoid the strong degradation of the ECE. It is known that low amounts (≤1 mol%) of Y_2_O_3_ addition act as Y^3+^ donors and lead to the formation of complex defects in the grain, which hinder abnormal grain growth. Further increasing the Y_2_O_3_ amount (>1 mol%) causes acceptor doping [17]. Depending on the sintering conditions, an addition of 0.625 mol% up to 1.25 mol% Y_2_O_3_ leads to the inhibition of grain growth due to the accumulation of Y^3+^ ions in the grain boundaries [17,18]. MnCO_3_ can also be used to suppress the grain growth through accumulation at the grain boundaries [19,20]. In addition, the modification of BSSnT with Ca^2+^ ions is also feasible, since an increasing Ca content in Ba_1−x_Ca_x_TiO_3_ (0 ≤ *x* ≤ 0.25) results in a remarkably decreased grain size (from 62 µm to 7 µm) [21].

In the present work, we study the influence of the grain-growth inhibitors MgO, Y_2_O_3_ and MnCO_3_ as well as Ca modification on the microstructure of BSSnT. For all samples, the resulting dielectric, ferroelectric and EC properties are characterized. Furthermore, the effects of the sintering time and temperature on the grain size and thus the EC properties of the promising material system Ba_0.62_Ca_0.20_Sr_0.18_Sn_0.065_Ti_0.935_O_3_ (BCSSnT-20) are investigated. The overall aim is to develop a lead-free material system that can be used in MLCs for future EC cooling systems.

## 2. Materials and Methods

Ba_0.82_Sr_0.18_Sn_0.065_Ti_0.935_O_3_ (BSSnT), Ba_0.72_Ca_0.10_Sr_0.18_Sn_0.065_Ti_0.935_O_3_ (BCSSnT-10) and Ba_0.62_Ca_0.20_Sr_0.18_Sn_0.065_Ti_0.935_O_3_ (BCSSnT-20) powders were synthesized via conventional solid-state reactions using BaCO_3_ (Solvay Chimica Italia S.p.A., Rosignano Solvay, Italy, 99.86%), CaCO_3_ (Thermo Fischer Scientific, Karlsruhe, Germany, 99.95–100.05%), SrCO_3_ (Alfa Aesar, Karlsruhe, Germany, 99%), SnO_2_ (abcr, Karlsruhe, Germany 99.9%) and TiO_2_ (Venator Germany GmbH, Duisburg, Germany, 99.5%). Details on the preparation are described in Refs. [15,16]. After calcination, different ratios of the sintering additives MgO (Merk Group, Darmstadt, Germany, ≥99%) Y_2_O_3_ (ChemPUR, Karlsruhe, Germany, 99.999%) and MnCO_3_ (ChemPUR, Karlsruhe, Germany, 99.9+%) were added to the BSSnT powder at a mole fraction of *x_i_* in relation to the BSSnT ($x_{MgO}$ = 0.5, 1%; $x_{{Y_{2}O}_{3}}$ = 0.25, 0.5, 1%; $x_{{MnCO}_{3}}$ = 0.5, 1, 1.5, 2%, respectively) to achieve the suppression of grain growth during sintering. The preparation of disc samples is described in Ref. [16]. A variation of sintering time was only carried out on BCSSnT-20 samples to vary their average grain size. Characterization of the microstructure was performed using Field Emission Scanning Electron Microscopy (FESEM). For this purpose, preparation of the cross-section of samples was performed followed by mechanical polishing. To achieve the image contrast, we used the channeling contrast of secondary electrons.

Furthermore, we characterized samples via X-ray diffraction (XRD), as well as dielectric, ferroelectric and direct electrocaloric measurements. For details on the methods and equipment see Refs. [16,22].

## 3. Results and Discussion

### 3.1. Microstructural Analysis

**Figure 1:**
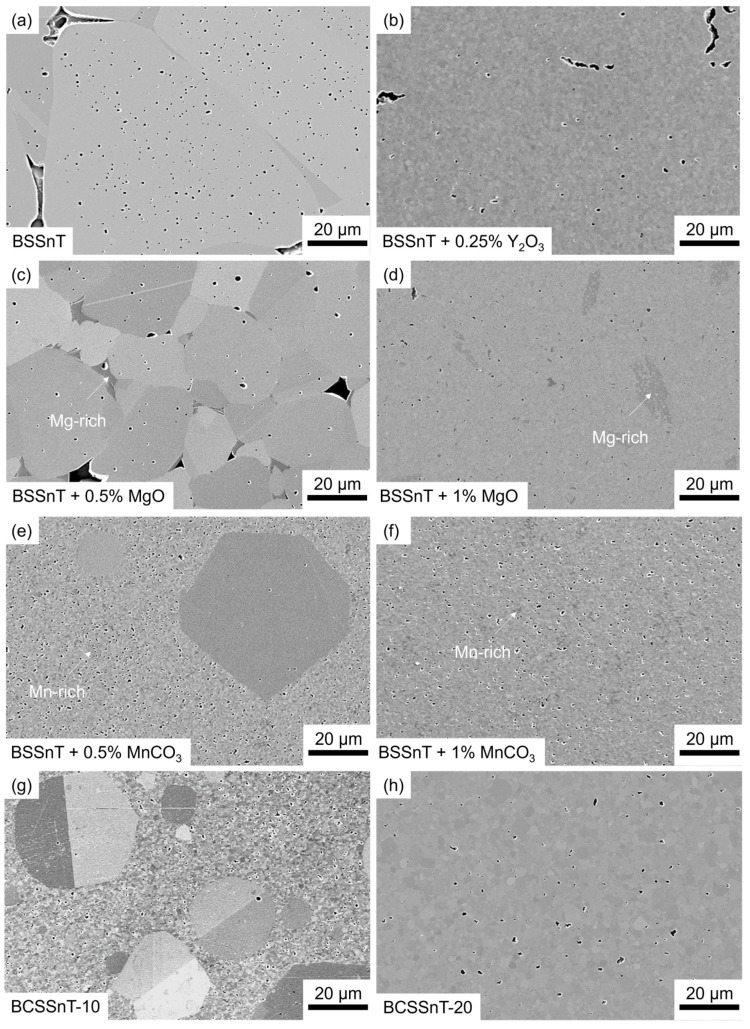
FESEM images (SE contrast) of (**a**) BSSnT; (**b**) BSSnT with the addition of Y_2_O_3_ ($x_{{Y_{2}O}_{3}}$ = 0.25%); (**c**) BSSnT with the addition of MgO ($x_{MgO}$ = 0.5%); (**d**) BSSnT with the addition of MgO ($x_{MgO}$ = 1%); (**e**) BSSnT with the addition of MnCO_3_ ($x_{{MnCO}_{3}}$ = 0.5%); (**f**) BSSnT with the addition of MnCO_3_ ($x_{{MnCO}_{3}}$ = 1%); (**g**) BCSSnT-10; and (**h**) BCSSnT-20.

Figure 1 shows the microstructure of the sintered bulk ceramics with different grain-growth inhibitors and Ca modifications. Compared to pure BSSnT with a mean grain size of around 40 µm (Figure 1a), the sintering additives Y_2_O_3_ ($x_{{Y_{2}O}_{3}}$ = 0.25%), MgO ($x_{MgO}$ = 1%) and MnCO_3_ ($x_{{MnCO}_{3}}$ = 1%) significantly decreased the mean grain size to 0.8 µm, 0.4 µm and 0.4 µm, respectively (Figure 1b,d,f). When adding MgO or MnCO_3_, Mg^2+^ or Mn^2+^/ Mn^3+^ ions predominantly precipitate at the grain boundaries, leading to the suppression of grain growth [19,20,23]. Too small an addition of MgO ($x_{MgO}$ = 0.5%) (Figure 1c) or MnCO_3_ ($x_{{MnCO}_{3}}$ = 0.5%) (Figure 1e) results in a coarsened microstructure or in the formation of individual coarse grains (>10 µm), which prevents the future fabrication of MLC components. In the BSSnT samples prepared with the addition of MgO or MnCO_3_, Mg-rich phases (Figure 1d) and Mn-rich phases (Figure 1f) were observed. These secondary phases already occurred at low contents of MgO (Figure 1c) or MnCO_3_ (Figure 1e). When using Y_2_O_3_, Y^3+^ ions accumulate at the grain boundary, forming core–shell structures and thus inhibiting further grain growth [24].

As shown in Figure 1g, samples prepared by the chemical modification of BSSnT with 10% Ca^2+^ on the A-site (BCSSnT-10) showed a bimodal grain size distribution consisting of fine grains (~0.6 µm) and giant grains (~21 µm), which is disadvantageous for MLC components. By increasing the amount of Ca^2+^ to 20% (BCSSnT-20), samples exhibited a homogeneous fine-grained microstructure with an average grain size of 1.5 µm (Figure 1h).

**Figure 2:**
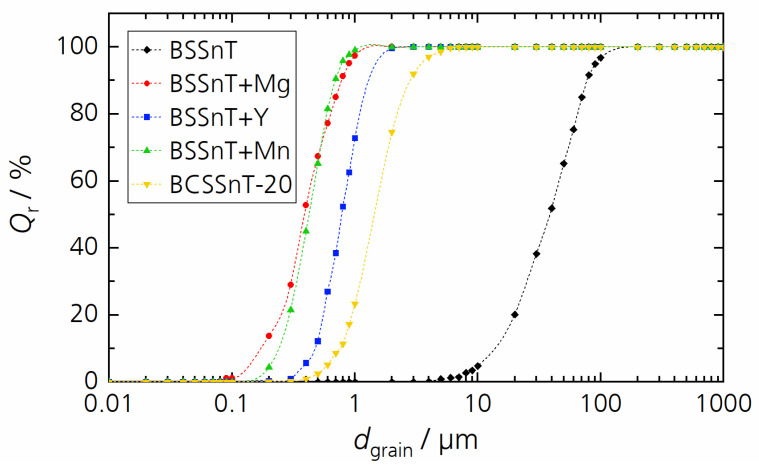
Cumulative distribution *Q*_r_ of grain size *d*_grain_ for sintered samples BSSnT (black), BSSnT + Mg (red), BSSnT + Y (blue), BSSnT + Mn (green) and BCSSnT-20 (yellow).

For the further investigation of the phase composition and electrical properties, only sintered bulk ceramics with a monomodal distribution of fine grains were selected and are summarized in Table 1 (BSSnT for reference). Their monomodal grain size distribution curves are shown in Figure 2.

### 3.2. Phase Anaylsis

**Figure 3:**
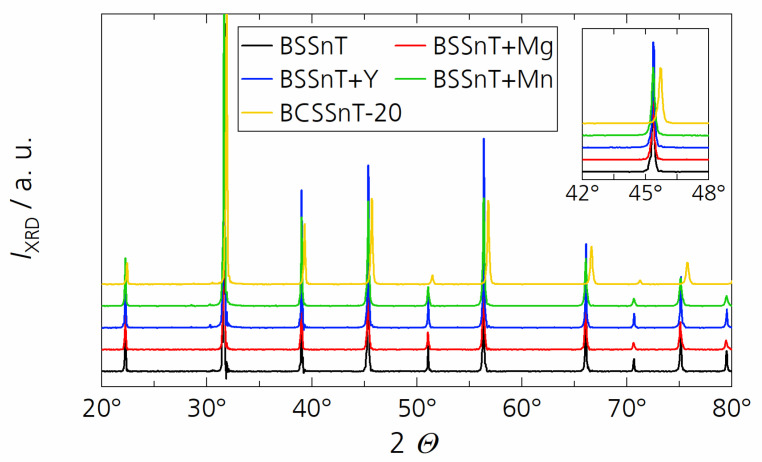
X-ray diffraction patterns of BSSnT (black), BSSnT + Mg (red), BSSnT + Y (blue), BSSnT + Mn (green) and BCSSnT-20 (yellow). (Inset: magnification between 42° and 48°).

The XRD patterns shown in Figure 3 indicate that pure perovskite phases were formed within the bulk ceramic samples with no occurrence of secondary phases. According to the Inorganic Crystal Structure Database (ICSD), the XRD pattern of all samples except BCSSnT-20 fit with the comparable material composition of Ba_0.6_Sr_0.39_Sn_0.26_Ti_0.74_O_3_ (ICSD file no. 01-077-9432), indicating a cubic crystal structure (Pm3m, 211 space group, *a* = 3.985 Å). The XRD pattern of BCSSnT-20 is in accordance with the peaks of the material composition of Ba_0.6_Sr_0.4_TiO_3_ (ICSD file no. 00-034-0411) with a cubic crystal structure (Pm3m, 211 space group, *a* = 3.965 Å). The inset of Figure 3 shows the XRD peaks at 2θ values between 42° and 48° for all samples. The modification of BSSnT with calcium caused a shift of the reflections in BCSSnT-20 to higher diffraction angles. These shifts can be explained by the substitution of Ba^2+^ ions (ionic radius: 175 pm) by Ca^2+^ ions (ionic radius: 148 pm) [25], which leads to a decrease in the lattice parameter and thus to higher diffraction angles.

### 3.3. Dielectric Properties

**Figure 4:**
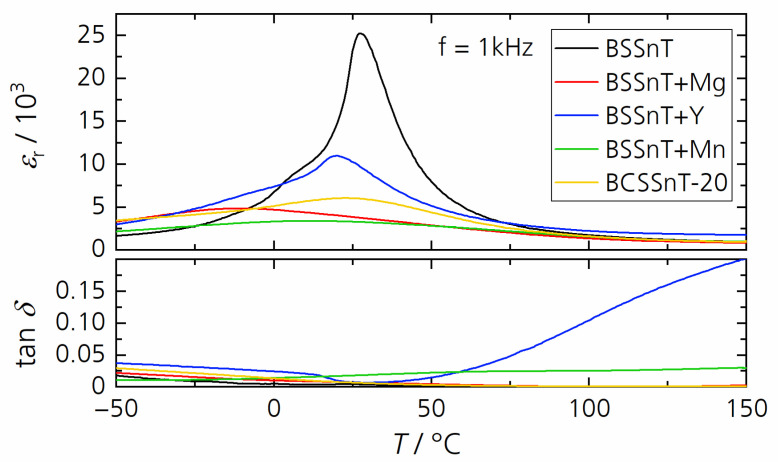
Temperature dependence of the relative permittivity *ε*_r_ and dielectric loss factor tan *δ* measured at 1 kHz for BSSnT (black), BSSnT + Mg (red), BSSnT + Y (blue), BSSnT + Mn (green) and BCSSnT-20 (yellow).

Figure 4 shows the relative permittivity and dielectric loss factor as functions of the temperature measured at 1 kHz for the investigated samples. Compared with BSSnT with a maximum relative permittivity of *ε*_r,m_ = 25,300 at *T*_m_ = 27 °C, all grain-growth inhibitors and the Ca modification significantly reduced the relative permittivity and shifted the temperature of the maximum permittivity towards lower temperatures with varying amplitudes. Among them, BCSSnT-20 had a permittivity peak of *ε*_r,m_ = 6000 at a slightly shifted temperature of *T*_m_ = 23 °C. In addition, the addition of MnCO_3_ resulted in an increased dielectric loss factor. This can be explained by the valence transformation of some manganese ions from Mn^2+^ to Mn^3+^ after sintering in air. The electrons released by the transformation increase the electrical conductivity and thus contribute to dielectric losses [26]. By contrast, the sharply increasing dielectric loss above 50 °C when adding Y_2_O_3_ to BSSnT is due to the formation of oxygen vacancies [27]. The temperature dependence of relative permittivity and the dielectric loss factor at different frequencies for each investigated composition are shown in Figure S1.

**Figure 5:**
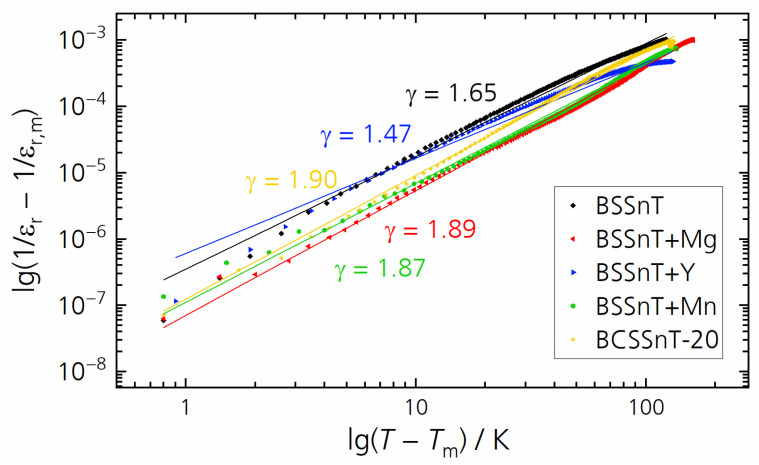
Plots of $\log\left(1/\varepsilon_{r}-1/\varepsilon_{r,m}\right)$ versus $\log\left(T-T_{m}\right)$ and fitted curves at 1 kHz for BSSnT (black), BSSnT + Mg (red), BSSnT + Y (blue), BSSnT + Mn (green) and BCSSnT-20 (yellow).

The diffuseness parameter of the phase transition can be determined using the following equation introduced by Uchino and Nomura [28]:

(1) $$\frac{1}{\varepsilon_{r}}-\frac{1}{\varepsilon_{r,m}}=\frac{{\left(T-T_{m}\right)}^{\gamma }}{C},\left(T>T_{m}\right),$$

where *C* is the Curie–Weiss constant, *γ* is the diffuseness coefficient with a value between 1 (for an ideal ferroelectric material) and 2 (for an ideal relaxor ferroelectric material). The plots of $\log\left(1/\varepsilon_{r}-1/\varepsilon_{r,m}\right)$ versus $\log\left(T-T_{m}\right)$ and their fitting curves at 1 kHz for the investigated samples are shown in Figure 5. The values of *γ* are in the range of 1.47–1.90, which corresponds to the diffuse phase transition caused by the existence of different states of polarization and hence different relaxation times in different regions [29]. The dielectric data of the investigated samples are summarized in Table 2. The addition of grain-growth inhibitors and Ca modification reduced the dielectric permittivity due to the decreased grain size. With the exception of the Y addition, the investigated grain-growth inhibitors showed no significant influence on the diffuseness of the phase transition.

### 3.4. Ferroelectric Properties

**Figure 6:**
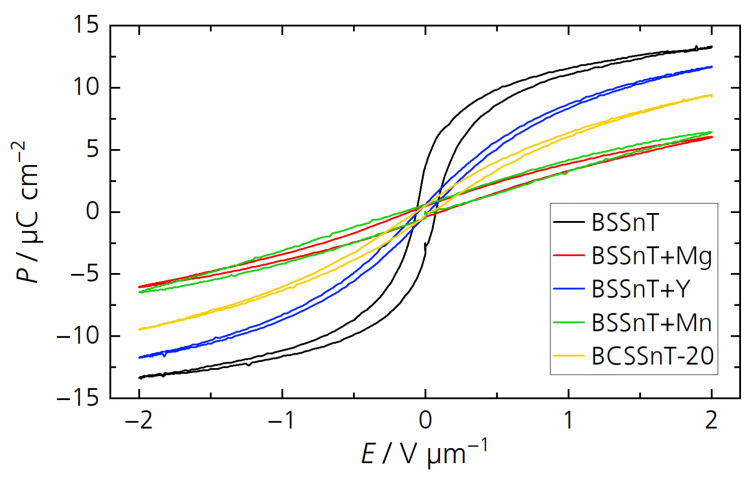
Hysteresis loops of polarization *P* in dependence of the electric field *E* for BSSnT (black), BSSnT + Mg (red), BSSnT + Y (blue), BSSnT + Mn (green) and BCSSnT-20 (yellow) measured at 20 °C and 10 Hz.

Hysteresis loops of polarization in dependence of the electric field varying from −2 to 2 V µm^−1^ measured at 20 °C and 10 Hz are presented in Figure 6. The samples prepared using grain-growth inhibitors showed a decreased maximum polarization *P*_m_ and a decreased remanent polarization *P*_r_ compared to pure BSSnT, which is related to their reduced grain size [30]. The addition of MgO to BSSnT led to a 55% decrease in the maximum polarization from *P*_m_ = 13.4 µC cm^−2^ to *P*_m_ = 6.1 µC cm^−2^, whereas the modification of BSSnT with Ca only reduced the maximum polarization by 30% to *P*_m_ = 9.5 µC cm^−2^. BCSSnT-20 therefore appears to be qualified for future multilayer ceramic fabrication. The ferroelectric characteristics of all studied compositions are summarized in Table 3.

### 3.5. Electrocaloric Properties

**Figure 7:**
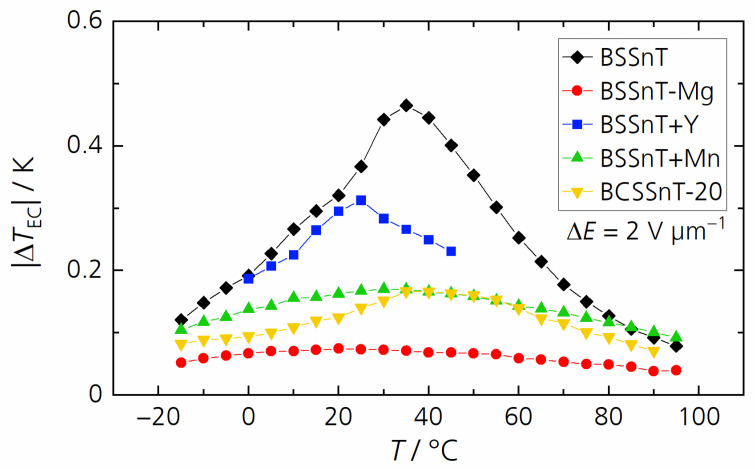
Electrocaloric temperature change |Δ*T*_EC_| depending on temperature *T* for BSSnT (black), BSSnT + Mg (red), BSSnT + Y (blue), BSSnT + Mn (green) and BCSSnT-20 (yellow) measured at Δ*E* = 2 V µm^−1^.

**Figure 8:**
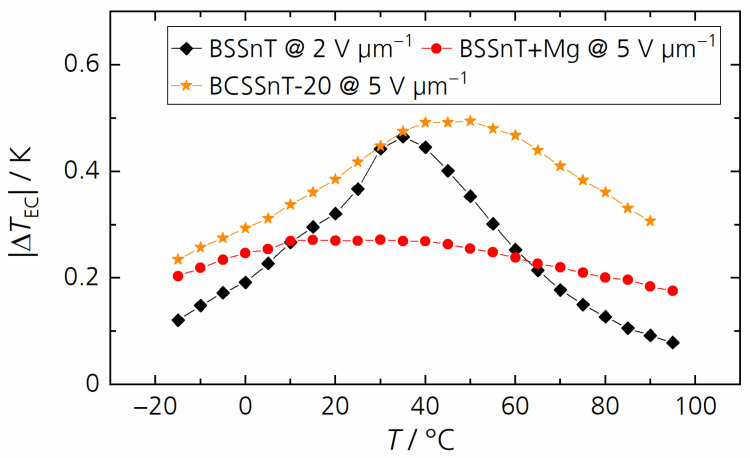
Electrocaloric temperature change |Δ*T*_EC_| depending on the temperature *T* for BSSnT (black) measured at Δ*E* = 2 V µm^−1^, and BSSnT + Mg (red) and BCSSnT-20 (yellow) measured at Δ*E* = 5 V µm^−1^.

Figure 7 shows the electrocaloric temperature change in dependence of the temperature measured at a relatively low electric field change of 2 V µm^−1^ for all samples. Compared to BSSnT featuring a maximum electrocaloric temperature change of 0.47 K at 35 °C, BSSnT + Y reached its maximum electrocaloric temperature change of 0.31 K at 25 °C. Measurement above 45 °C was not possible for this composition because of the high dielectric losses. BSSnT-Mg, BSSnT-Mn and BCSSnT-20 showed a lower EC effect over a broadened temperature range. However, BSSnT-Mg and BCSSnT-20 can withstand higher electric field changes during EC measurements. Under the application of an electric field change of 5 V µm^−1^ BSSnT-Mg showed an electrocaloric temperature change of 0.27 K in a broad temperature range from 5 to 50 °C, while BCSSnT-20 showed a maximum electrocaloric temperature change of 0.49 K at 40 °C with a broad peak of |Δ*T*_EC_| > 0.33 K in the temperature range from 10 °C to 75 °C (Figure 8). The results indicate that BCSSnT-20 is most suitable for the future fabrication of MLC components, where the application of higher electrical fields and thus higher EC effects are expected.

### 3.6. Influence of Sintering Time and Temprature on BCSSnT-20 Bulk Ceramics

Since the EC effect is not only affected by the applied electrical field but also determined by the grain size of the samples [31,32], we further studied this correlation on the material BCSSnT-20. Therefore, the microstructure of samples with the same chemical composition was modified by variations of sintering time and temperature.

**Figure 9:**
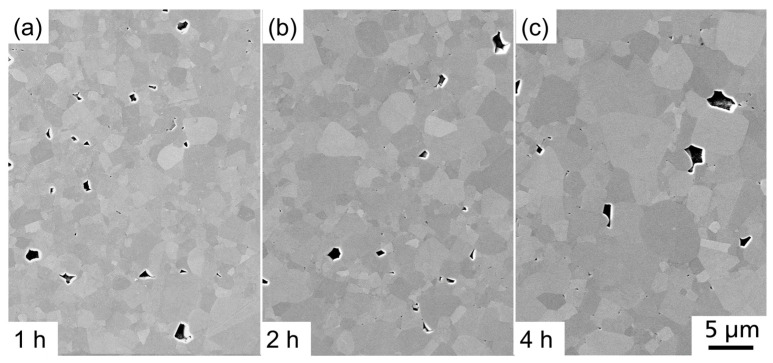
FESEM images (SE contrast) of BCSSnT-20 sintered at 1350 °C for (**a**) 1 h; (**b**) 2 h; and (**c**) 4 h.

**Figure 10:**
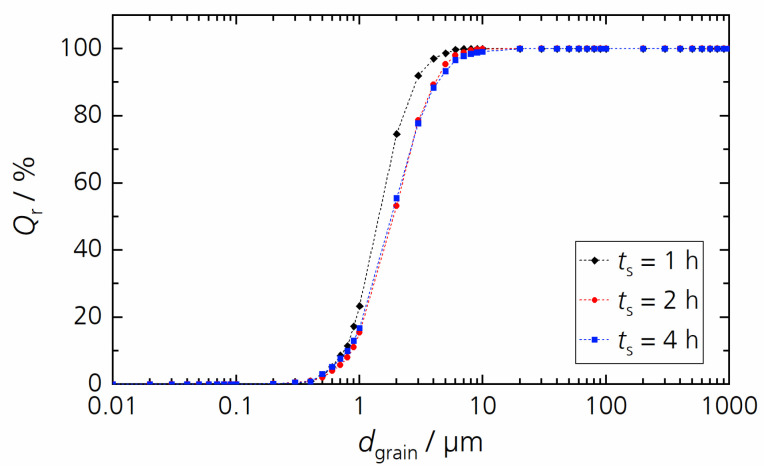
Cumulative distribution *Q*_r_ of grain size *d*_grain_ for BCSSnT-20 sintered at 1350 °C for 1 h (black), 2 h (red) and 4 h (blue).

Our variations of sintering time with a constant sintering temperature of 1350 °C only slightly influenced the microstructure of BCSSnT-20 (shown in Figure 9). With an increase in sintering time from 1 h to 4 h, the grain size increased from 1.5 µm to 1.9 µm (Figure 10), accompanied by an increase in porosity from 0.6% to 1.0%.

**Figure 11:**
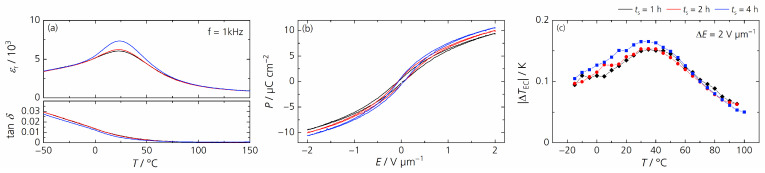
(**a**) The temperature dependence of the relative permittivity *ε*_r_ and dielectric loss factor tan *δ* measured at 1 kHz; (**b**) hysteresis loops of the polarization *P* in dependence of the electric field *E* measured at 20 °C and 10 Hz; (**c**) the electrocaloric temperature change |Δ*T*_EC_| depending on temperature *T* for BCSSnT-20 sintered at 1350 °C for 1 h (black), 2 h (red) and 4 h (blue).

The electrical properties were marginally improved by increasing the sintering time (Figure 11). The maximum relative permittivity rose negligibly with *T*_m_ remaining unchanged. The slim hysteresis loops indicate that the samples showed relaxor-like behavior, and the temperature-dependent behavior of the remanent polarization indicates that the samples underwent a diffuse phase transition. In addition, the remanent polarization increased faintly with increasing sintering time and so did the electrocaloric temperature change. Results of the characterization are summarized in Table 4 (for the relative permittivity *ε*_r_ measured at different frequencies, the corresponding diffuseness coefficient *γ* and the temperature-dependent remanent polarization, see Figures S2–S4.) In conclusion, varying the sintering time affected the grain size slightly and thus only moderately changed the electrocaloric properties.

**Figure 12:**
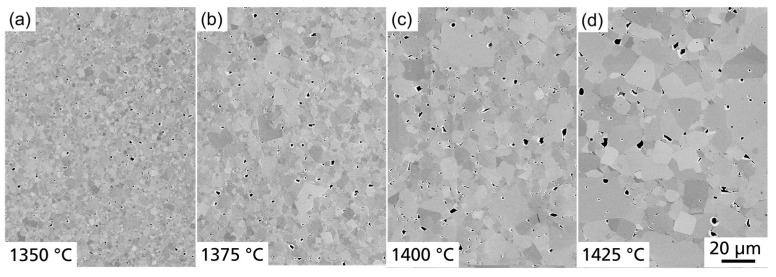
FESEM images (SE contrast) of BCSSnT-20 sintered for 1 h at (**a**) 1350 °C; (**b**) 1375 °C; (**c**) 1400 °C; and (**d**) 1425 °C.

**Figure 13:**
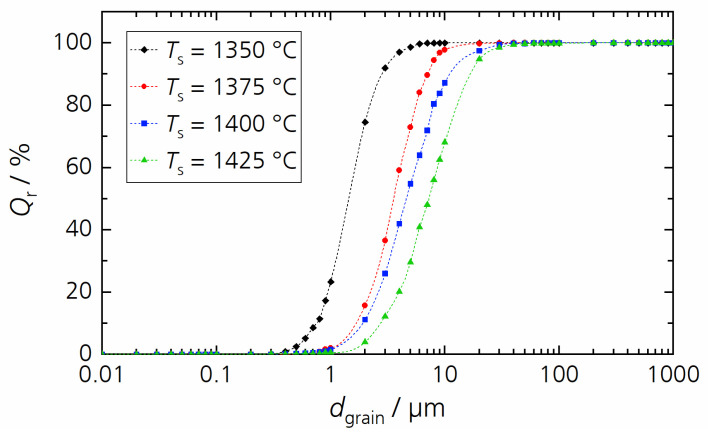
The cumulative distribution *Q*_r_ of grain size *d*_grain_ for BCSSnT-20 sintered for 1 h at 1350 °C (black), 1375 °C (red), 1400 °C (blue) and 1425 °C (red).

**Figure 14:**
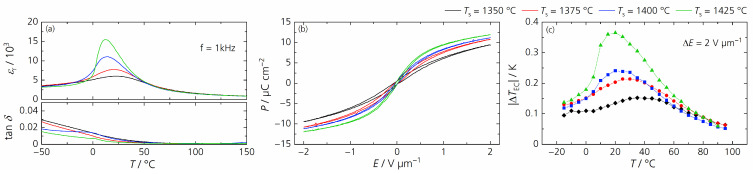
(**a**) Temperature dependence of the relative permittivity *ε*_r_ and dielectric loss factor tan *δ* measured at 1 kHz; (**b**) hysteresis loops of the polarization *P* in dependence of the electric field *E* measured at 20 °C and 10 Hz; (**c**) the electrocaloric temperature change |Δ*T*_EC_| depending on the temperature *T* for BCSSnT-20 sintered for 1 h at 1350 °C (black), 1375 °C (red), 1400 °C (blue) and 1425 °C (green).

In the second step, we investigated the influence of the sintering temperature *T*_S_ with a constant sintering time of 1 h. Figure 12 presents the microstructure and Figure 13 the grain size distribution of BCSSnT-20 sintered at sintering temperatures up to 1425 °C. Increasing the sintering temperature led to a grain coarsening from 1.5 µm at 1350 °C to 3.6 µm at 1375 °C, 4.7 µm at 1400 °C and up to 7.3 µm at 1425 °C. Simultaneously, the porosity increased from 0.6% to 2.2%. Along with the increase in the sintering temperature and grain size, the maximum relative permittivity increased significantly with a shift of the temperature of maximum permittivity towards lower temperatures (shown in Figure 14a). Moreover, the peak of relative permittivity became shaper and the diffuseness coefficient *γ* decreased from 1.90 to 1.72, indicating that the phase transition is no longer suppressed and thus less diffuse in samples with coarse grains [33]. The remanent polarization increased with increasing sintering temperature and grain size. With an increasing grain size, the number of low-permittivity grain boundaries is reduced, resulting in less polarization discontinuity on the grain surface and thus improved polarization [34]. The maximum electrocaloric temperature change |Δ*T*_EC_| increased from 0.15 K at 35 °C to 0.37 K at 20 °C under an electric field change of 2 V µm^−1^, which can also be explained by the dependency of the EC effect on the grain size [35,36,37]. Results of the characterization of the BCSSnT-20 samples in dependence of their sintering temperature are summarized in Table 5 (for the relative permittivity *ε*_r_ measured at different frequencies, the corresponding diffuseness coefficient *γ* and the temperature-dependent remanent polarization see Figures S5–S7.) Additionally, the typical values of the directly measured electrocaloric temperature change published for selected BaTiO_3_-based materials are shown in Table 6. The sample of BCSSnT-20 sintered at 1425 °C was in good agreement with previously published results, exhibiting an electrocaloric strength of 0.19 10^−6^ K m V^−1^. Simultaneously, the maximum of the ECE occurred around room temperature.

In summary, increasing the sintering temperature affected the grain size significantly and thus enhanced the electrocaloric properties of the ceramic samples. However, the dielectric breakdown strength of ceramics decreases with increasing grain size, for example in barium titanate with the relation $E\propto G^{-a}$, where *E*, *G* and *a* are the breakdown field strength, the grain size and a constant of approximately 0.5, respectively [38]. Therefore, thorough grain-size engineering aiming at high dielectric strength and high EC properties will be necessary for future MLC components.

**Table 6:** Compilation of the directly measured electrocaloric temperature change (|Δ*T*_EC_|) at different electric field changes (Δ*E*), as well as the corresponding temperature (*T*) and electrocaloric strength (Δ*T*/Δ*E*) for the selected BaTiO_3_-based materials.

Material	*T*/ °C	Δ*E*/ V µm^−1^	|Δ*T*_EC_|/ K	Δ*T*/Δ*E*/ 10^−6^ K m V^−1^	Measurement	Ref.
Ba_0.62_Ca_0.20_Sr_0.18_Sn_0.065_Ti_0.935_O_3_	20	2	0.37	0.19	thermocouple	This work
(Ba_0.8_Ca_0.2_)_1−x_La_2x/3_TiO_3_	77	2.5	0.3	0.12	calorimeter	[39]
Ba_0.95_Ca_0.05_Ti_0.92_Sn_0.08_O_3_	55	2	0.32	0.16	DSC	[40]
Ba_1−x_Ca_x_Ti_0.9_Zr_0.1_O_3_	90	1.6	0.29	0.18	Pt100	[41]
Ba_0.9_Sr_0.1_Ti_0.95_Sn_0.05_O_3_	67	0.7	0.19	0.27	thermistor	[42]
Ba_0.77_Ca_0.21_Sn_0.02_Ti_0.94_Nb_0.04_Sn_0.02_O_3_	17	5	0.18	0.04	thermistor	[43]
Ba_0.87_Ca_0.13_Ti_0.87_Hf_0.13_O_3_	65	1	0.30	0.30	DSC	[44]
Ba_0.82_Sr_0.18_Sn_0.065_Ti_0.935_O_3_	31	2	0.49	0.25	thermocouple	[15]
(Ba_0.82_Sr_0_._18_)_x_(Sn_0.65_Ti_0.935_)_y_O_3_	30	5	0.59	0.12	thermoelement	[22]
Ba_1−y_Ca_y_Ti_1−x_Hf_x_O_3_	57	1.6	0.20	0.13	thermocouple	[45]

## 4. Conclusions

In the present work, we investigate the influence of the grain-growth inhibitors MgO, Y_2_O_3_ and MnCO_3_ as well as Ca modification on the microstructure, electrical properties and electrocaloric characteristics of BSSnT bulk ceramic samples. The addition of MgO ($x_{MgO}$ = 1%), Y_2_O_3_ ($x_{{Y_{2}O}_{3}}$ = 0.25%) and MnCO_3_ ($x_{{MnCO}_{3}}$ = 1%) significantly decreased the average grain size of BSSnT from 40 µm to 0.4 µm, 0.8 µm and 0.4 µm, respectively. However, the additions of Y_2_O_3_ and MnCO_3_ caused high dielectric losses, especially at elevated temperatures, making the resulting material inappropriate for a future fabrication of EC multilayer ceramic components. The same applies to BSSnT modified with 10% of Ca^2+^ (BCSSnT-10), which exhibited a bimodal grain size distribution with coarse grains (~21 µm). In comparison, the modification of BSSnT with 20% Ca^2+^ (BCSSnT-20) showed an average grain size of 1.5 µm and a maximum EC temperature change |Δ*T*_EC_| of 0.49 K at 40 °C with a broad peak of |Δ*T*_EC_| > 0.33 K in the temperature range from 10 °C to 75 °C under an electric field change of 5 V µm^−1^. Since the EC effect is also affected by the grain size of the samples, additional sintering experiments were performed on BCSSnT-20. By increasing the sintering temperature from 1350 °C to 1425 °C, the grain size was increased from 1.5 µm to 7.3 µm and the maximum electrocaloric temperature change |Δ*T*_EC_| was enhanced from 0.15 K to 0.37 K under an electric field change of 2 V µm^−1^. Our results show that among all investigated material systems, BCSSnT-20 is a very promising candidate for application in multilayer ceramic (MLC) components for EC cooling devices. Future work will concentrate on the preparation of BCSSnT-20 MLC components, grain size engineering, and the correlation between the microstructure, dielectric strength, and EC properties.

## Figures and Tables

**Table 1:** Compilation of samples prepared with different mole fractions of additives (*x*_additive_). Sintering temperature (*T*_S_) and average grain size (*d*_50_) refer to emphasized concentrations (bold font), which were also used for further investigations.

Sample	Additive	*x*_additive_/%	*T*_S_/°C	*d*_50_/µm
BSSnT	-	-	1400	40
BSSnT + Mg	MgO	0.5, **1**	1400	0.4
BSSnT + Y	Y_2_O_3_	**0.25**, 0.5, 1	1350	0.8
BSSnT + Mn	MnCO_3_	0.5, **1**, 1.5, 2	1350	0.4
BCSSnT-20	CaCO_3_	10, **20**	1350	1.5

**Table 2:** Compilation of the temperature of maximum permittivity (*T*_m_), the maximum relative permittivity (*ε*_r,m_), the loss factor at *T*_m_ (tan *δ*_m_) with standard deviations and the diffuseness coefficient (*γ*) with standard deviations of BSSnT, BSSnT + Mg, BSSnT + Y, BSSnT + Mn and BCSSnT-20 (measured at 1 kHz).

Sample	*T*_m_/°C	*ε*_r,m_	tan *δ*_m_	*γ*
BSSnT	27	25,300	0.0041(1)	1.65 (6)
BSSnT + Mg	−11	4900	0.0119(1)	1.89 (9)
BSSnT + Y	20	11,000	0.0111(3)	1.47 (2)
BSSnT + Mn	14	3400	0.0158(1)	1.87 (5)
BCSSnT-20	23	6000	0.0074(2)	1.90 (4)

**Table 3:** Compilation of the maximum polarization (*P*_m_), remanent polarization (*P*_r_) and coercive field *E*_c_ for BSSnT, BSSnT + Mg, BSSnT + Y, BSSnT + Mn and BCSSnT-20 (measured at 10 Hz, 2 V µm^−1^).

Sample	*P*_m_/µC cm^−2^	*P*_r_/µC cm^−2^	*E*_c_/10^−2^ V µm^−1^
BSSnT	13.4	3.3	4.7
BSSnT + Mg	6.1	0.4	8.6
BSSnT + Y	11.7	0.3	0.6
BSSnT + Mn	6.5	0.6	3.5
BCSSnT-20	9.5	0.5	1.6

**Table 4:** Compilation of the average grain size (*d*_50_) and porosity (*Φ*); the temperature of maximum permittivity (*T*_m_), maximum relative permittivity (*ε*_r,m_), loss factor at *T*_m_ (tan *δ*_m_) with standard deviations and diffuseness coefficient (*γ*) with standard deviations measured at 1 kHz; the maximum polarization (*P*_m_), remanent polarization (*P*_r_) and coercive field *E*_c_ measured at 20 °C and 10 Hz; the maximum electrocaloric temperature change (|Δ*T*_EC_|)measured at an electric field change of 2 V µm^−1^; and the corresponding temperature (*T*) for BCSSnT-20 sintered at 1350 °C with sintering times (*t*_s_) of 1 h, 2 h and 4 h.

*t*_s_/ h	*d*_50_/ µm	*Φ*/ %	*T*_m_/ °C	*ε*_r,m_	tan *δ*_m_	*γ*	*P*_m_/ µC cm^−2^	*P*_r_/ µC cm^−2^	*E*_c_/ 10^−2^ V µm^−1^	*T*/ °C	|Δ*T*_EC_|/ K
1	1.5	0.6	23	6000	0.0074 (2)	1.90 (4)	9.5	0.5	1.6	35	0.15
2	1.9	0.7	23	6200	0.0068 (2)	1.93 (8)	10.1	0.6	5.5	35	0.15
4	1.9	1.0	23	7300	0.0055 (2)	1.89 (7)	10.6	0.6	2.2	35	0.17

**Table 5:** Compilation of the average grain size (*d*_50_) and porosity (*Φ*); the temperature of maximum permittivity (*T*_m_), maximum relative permittivity (*ε*_r,m_), loss factor at *T*_m_ (tan *δ*_m_)with standard deviations and diffuseness coefficient (*γ*) with standard deviations measured at 1 kHz; the maximum polarization (*P*_m_), remanent polarization (*P*_r_) and coercive field (*E*_c_) measured at 20 °C and 10 Hz; the maximum electrocaloric temperature change (|Δ*T*_EC_|) measured at an electric field change of 2 V µm^−1^; and the corresponding temperature (*T*) for BCSSnT-20 sintered for 1 h at sintering temperatures (*T*_s_) of 1350 °C, 1375 °C, 1400 °C and 1425 °C.

*T*_s_/ °C	*d*_50_/µm	*Φ*/ %	*T*_m_/ °C	*ε*_r,m_	tan *δ*_m_	*γ*	*P*_m_/ µC cm^−2^	*P*_r_/ µC cm^−2^	*E*_c_/ 10^−2^ V µm^−1^	T/ °C	|Δ*T*_EC_|/ K
1350	1.5	0.6	23	6000	0.0074 (2)	1.90 (4)	9.5	0.5	1.6	35	0.15
1375	3.6	1.2	20	7800	0.0045 (1)	1.86 (2)	10.8	0.5	2.0	25	0.21
1400	4.7	2.4	13	11,000	0.0080 (10)	1.77 (6)	11.4	0.4	0.2	20	0.24
1425	7.3	2.2	13	15,000	0.0045 (9)	1.72 (5)	12.0	0.2	0.1	20	0.37

## Data Availability

Data are contained within the article or Supplementary Materials.

## Supplementary Materials

The following supporting information can be downloaded at https://www.mdpi.com/article/10.3390/ma17051036/s1: Figure S1. Temperature dependence of the relative permittivity *ε*_r_ and dielectric loss factor tan *δ* measured at different frequencies for (**a**) BSSnT; (**b**) BSSnT + Mg; (**c**) BSSnT + Y; (**d**) BSSnT + Mn; and (**f**) BCSSnT-20. Figure S2. Temperature dependence of the relative permittivity *ε*_r_ and dielectric loss factor tan *δ* measured at different frequencies for BCSSnT-20 sintered at 1350 °C for (**a**) 1 h; (**b**) 2 h; and (**c**) 4 h. Figure S3. Plots of $\log\left(1/\varepsilon_{r}-1/\varepsilon_{r,m}\right)$ versus $\log\left(T-T_{m}\right)$ and fitted curves at 1 kHz for BCSSnT-20 sintered at 1350 °C for 1 h (black), 2 h (red) and 4 h (blue). Figure S4. Temperature-dependent measurement of the remanent polarization *P*_r_ for BCSSnT-20 sintered at 1350 °C for 1 h (black), 2 h (red) and 4 h (black) measured at 2 V µm^−1^ and 10 Hz. Figure S5. Temperature dependence of the relative permittivity *ε*_r_ and dielectric loss factor tan *δ* measured at different frequencies for BCSSnT-20 for 1 h at (**a**) 1350 °C; (**b**) 1375 °C; (**c**) 1400 °C; and (**d**) 1425 °C. Figure S6. Plots of $\log\left(1/\varepsilon_{r}-1/\varepsilon_{r,m}\right)$ versus $\log\left(T-T_{m}\right)$ and fitted curves at 1 kHz for BCSSnT-20 sintered for 1 h at 1350 °C (black), 1375 °C (red), 1400 °C (blue) and 1425 °C (green). Figure S7. Temperature-dependent measurement of the remanent polarization *P*_r_ for BCSSnT-20 sintered for 1 h at 1350 °C (black), 1375 °C (red), 1400 °C (blue) and 1425 °C (green) measured at 2 V µm^−1^ and 10 Hz.
