# Supplementary material for: Influence of Grain-Growth Inhibitors on Modified (Ba,Sr)(Sn,Ti)O3 for Electrocaloric Application
Source: Materials (Basel). 2024 Feb 23;17(5):1036. doi: 10.3390/ma17051036 (PMC10933918; doi:10.3390/ma17051036)
Supplement: Supplementary file 1 [file materials-17-01036-s001.zip › materials-2880114-supplementary.pdf]

materials

SUPPLEMANTARY

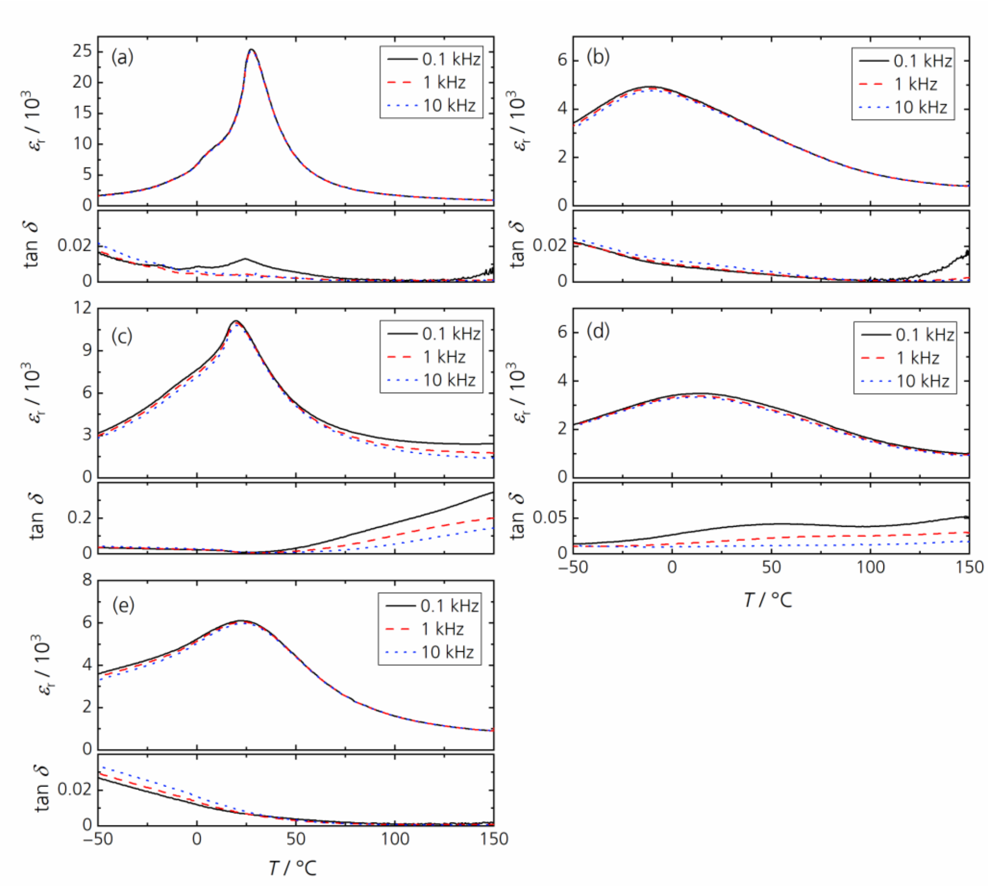

Figure S1. Temperature dependence of relative permittivity ε_r and dielectric loss factor tan δ measured at different frequencies for (**a**) BSSnT, (**b**) BSSnT+Mg, (**c**) BSSnT+Y, (**d**) BSSnT+Mn, and (**e**) BCSSnT-20.

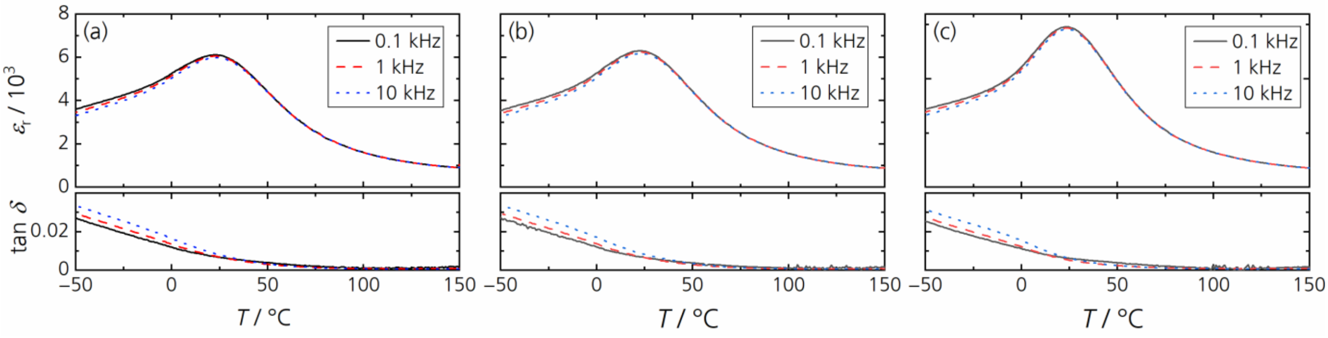

Figure S2. Temperature dependence of relative permittivity ε_r and dielectric loss factor tan δ measured at different frequencies for BCSSnT-20 sintered at 1350 °C for (**a**) 1 h, (**b**) 2 h and (**c**) 4 h.

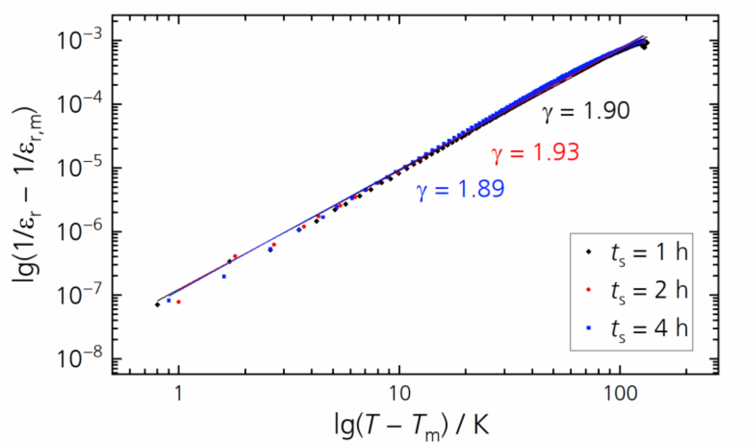

Figure S3. Plots of $\log(1/\varepsilon_r - 1/\varepsilon_{r,m})$ versus $\log(T - T_m)$ and fitted curves at 1 kHz for BCSSnT-20 sintered at 1350 °C for 1 h (black), 2 h (red) and 4 h (blue).

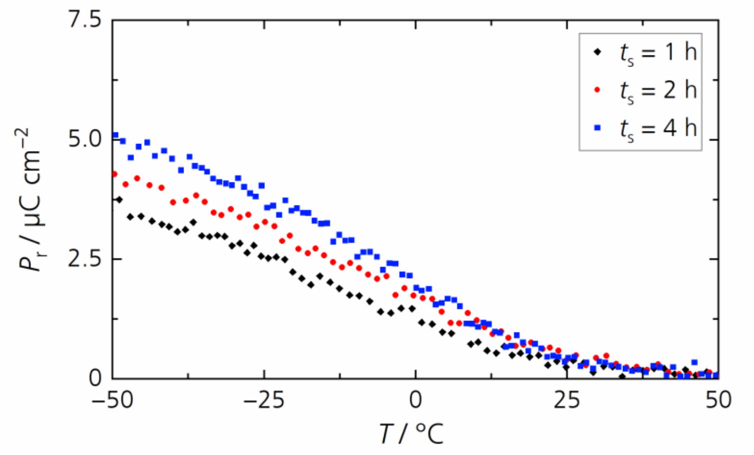

Figure S4. Temperature-dependent measurement of remanent polarization P_r for BCSSnT-20 sintered at 1350 °C for 1 h (black), 2 h (red) and 4 h (black) measured at 2 V µm⁻¹ and 10 Hz.

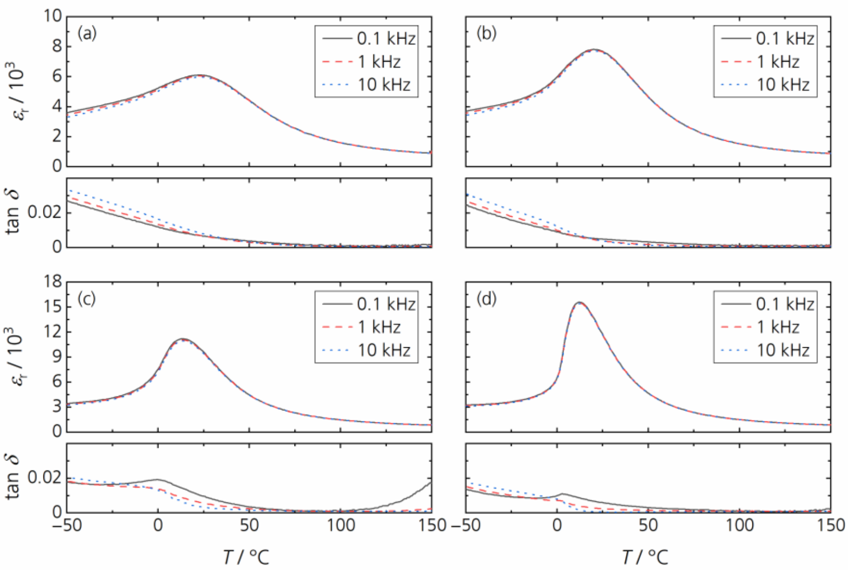

Figure S5. Temperature dependence of relative permittivity ε_r and dielectric loss factor $\tan\delta$ measured at different frequencies for BCSSnT-20 for 1 h at (**a**) 1350 °C, (**b**) 1375 °C, (**c**) 1400 °C and (**d**) 1425 °C.

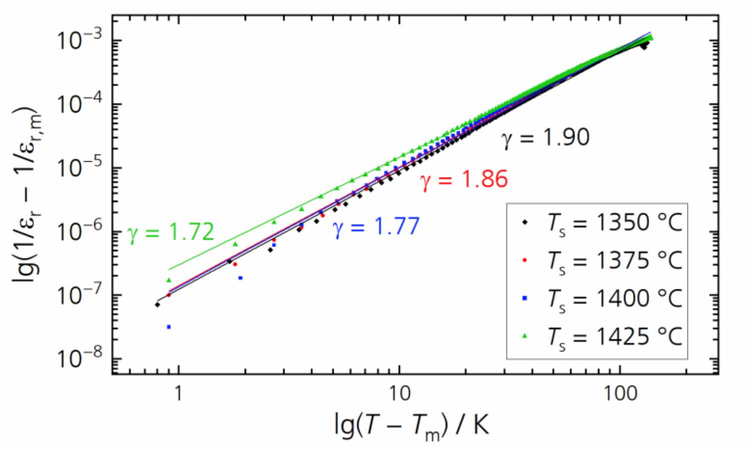

Figure S6. Plots of $\log(1/\varepsilon_r - 1/\varepsilon_{r,m})$ versus $\log(T - T_m)$ and fitted curves at 1 kHz for BCSSnT-20 sintered for 1 h at 1350 °C (black), 1375 °C (red), 1400 °C (blue) and 1425 °C (green).

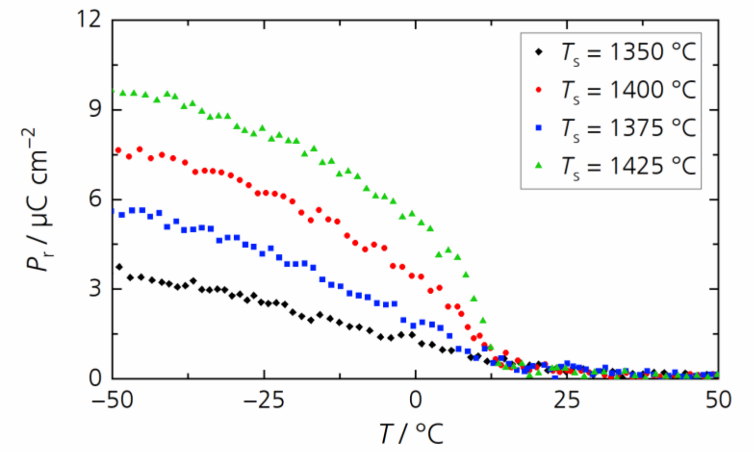

Figure S7. Temperature-dependent measurement of remanent polarization P_r for BCSSnT-20 sintered for 1 h at 1350 °C (black), 1375 °C (red), 1400 °C (blue) and 1425 °C (green) measured at 2 V μm^{-1} and 10 Hz.
